# Atypical Postural Control Variability and Coordination Persist Into Middle and Older Adulthood in Autism Spectrum Disorder

**DOI:** 10.1002/aur.70024

**Published:** 2025-03-18

**Authors:** Hang Qu, Jingying Wang, Desirae J. Shirley, Hanna M. Gemmell, Danielle Christensen, Ann‐Marie Orlando, Regilda A. Romero, Brandon A. Zielinski, Zheng Wang

**Affiliations:** ^1^ Neurocognitive and Behavioral Development Laboratory, Department of Applied Physiology and Kinesiology University of Florida Gainesville Florida USA; ^2^ Department of Applied Physiology and Kinesiology University of Florida Gainesville Florida USA; ^3^ Center for Autism and Related Disabilities (CARD) University of Florida Gainesville Florida USA; ^4^ UF Health Center for Autism and Neurodevelopment (UF Health CAN) University of Florida Gainesville Florida USA; ^5^ Department of Psychiatry University of Florida Gainesville Florida USA; ^6^ Department of Pediatrics, Neurology, and Neuroscience University of Florida Gainesville Florida USA; ^7^ Department of Psychology University of Florida Gainesville Florida USA; ^8^ Rehabilitation Science Program University of Florida Gainesville Florida USA

**Keywords:** aging, autism spectrum disorder, dynamic postural sway, middle aged and older, postural control, static stance

## Abstract

Postural control deviations remain largely unexplored in middle aged and older autistic adults. With the increased prevalence of neurodegenerative conditions and heightened fall risk, precise quantification of postural variability and coordination may provide valuable insights into aging associated neuromotor deviations in autistic adults. Forty‐seven autistic and 48 non‐autistic individuals completed static stance, anterior–posterior (AP), and mediolateral (ML) postural sway on a force platform. Center of pressure (COP) metrics were derived and interpreted using ANCOVAs for between‐group comparisons and multilinear regressions for group × age interaction. Correlations between clinical measures and COP variables that differentiated groups were explored. Compared to non‐autistic individuals, autistic adults exhibited greater COP standard deviation (COP_SD_) and COP trajectory length during static stance and demonstrated significant COP_SD‐AP_ reductions in older age. Autistic adults also exhibited decreased COP range of motion (ROM) but increased ROM variability in the target direction during dynamic stance. Autistic adults' postural sway was jerkier during dynamic stance, and increased ROM variability during dynamic AP sway was moderately associated with lower verbal IQ in autistic adults. Our findings highlight persistent postural control deviations in middle aged and older autistic adults. Static and dynamic stance are differentially associated with unique profiles of postural control in ASD. Specifically, autistic adults demonstrated pronounced increases in postural sway variability during static stance, while reducing coordination during dynamic conditions. The extent to which postural control deviations found in autistic adults are predictive to the onset of neurodegenerative conditions and the severity of falls warrants future longitudinal research.

**Abbreviations**
ADOS‐2: Autism Diagnostic Observation Schedule, second edition
AP: anterior–posterior direction
AQ: Autism Spectrum Quotient
ASD: Autism spectrum disorder
BMI: body mass index
CARD: Center for Autism and Related Disabilities
COP: center of pressure
COP_AP_
: COP time series in the anterior–posterior direction
COP_DSJP_
: COP dimensionless squared jerk normalized by peak velocity
COP_Freq_
: COP frequency (i.e., postural sway frequency)
COP_ML_
: COP time series in the mediolateral direction
COP_SD_
: standard deviation of COP time series
COP_SD‐AP_
: standard deviation of COP_AP_
COP_SD‐ML_
: standard deviation of COP_ML_
COP_TL_
: COP trajectory length
DSM‐5: Diagnostic and Statistical Manual of Mental Disorders, Fifth Edition
FDR: false discovery rate
fsIQ: full‐scale intelligence quotient
IRB: Institutional Review Board
ML: mediolateral direction
pIQ: performance intelligence quotient
RBS‐R: repetitive behavior scale‐revised
ROM: range of motion
ROM_avg_
: average COP range of motion in the target direction during a dynamic trial
ROM_cv_
: coefficient of variation of the COP range of motion in the target direction during a dynamic trial (i.e., the ratio of ROM_SD_ and ROM_avg_)
ROM_SD_
: standard deviation of the COP range of motion in the target direction during a dynamic trial
SRS‐2: Social Responsiveness Adult Self‐Report Scale, second edition
vIQ: verbal intelligence quotient
WASI‐II: Wechsler Abbreviated Scale of Intelligence, second edition

**Summary**

- We examined standing posture in 47 autistic and 48 non‐autistic adults aged 30–73 years to better understand if postural control impairments commonly found in autistic children extend into middle and older adulthood and how age affects balance in autistic adults.
- We found increased postural sway variability and less smooth (or jerkier) postural sway trajectories in autistic adults compared to non‐autistic adults.
- We will follow our participants over time to determine if aging accelerates postural control deviations in middle aged and older autistic adults.

## 1 Introduction

Neuromotor dysfunction is one of many common co‐occurring conditions in autism spectrum disorder (ASD), significantly impacting individuals' independence, community engagement, and quality of life (Bhat et al. 2012; Hannant et al. 2016; Jansiewicz et al. 2006; Mosconi and Sweeney 2015; Mosconi et al. 2015). Postural control, a fundamental motor skill, plays a critical role in maintaining torso orientation, enhancing mobility, and enabling purposeful movement (Horak 2006; Riccio 1993). While increased variability in postural sway and reduced coordination are consistently documented in autistic children and adolescents, postural control remains largely unexplored in middle aged and older autistic adults. Notably, epidemiological studies have identified an elevated risk of falls in ASD, which are the leading cause of physical injuries and emergency department visits among middle aged and older autistic individuals (Howlin 2023; Vohra et al. 2016). Additionally, autistic adults exhibit higher rates of metabolic and chronic illness, including obesity, type 2 diabetes, hypertension, and cardiovascular disease (Croen et al. 2015; Hand et al. 2020), all of which further exacerbate postural instability and increase fall risk. Given that postural control strongly predicts fall risk in elderly individuals (Dominguez 2020; Johansson et al. 2019; Melzer et al. 2004), a precise quantification of postural variability and coordination would provide valuable insights into the neurophysiological mechanisms underlying fall susceptibility in middle aged and older autistic adults. Such efforts would ultimately promote targeted clinical interventions to enhance balance and mobility in ASD.

Autistic children and adolescents show increased postural sway variability during static stance, particularly when sensory information is minimized or distorted (Gepner and Mestre 2002; Gepner et al. 1995; Kohen‐Raz et al. 1992; Minshew et al. 2004; Molloy et al. 2003; Morris et al. 2015). However, less empirical attention has been given to the assessment of dynamic stance, despite dynamic tasks outperforming static stance in predicting fall risk (Horak et al. 1997; Ringhof and Stein 2018; Rizzato et al. 2021). Static and dynamic stance differ in both task goals and neurophysiological mechanisms that underlie performance. During static stance, individuals minimize postural sway variability to enhance stability (Horak 2006; Morris et al. 2015; Riccio 1993; Wang et al. 2016). In contrast, during dynamic stance, individuals intentionally shift their postural sway toward the postural limitation boundary to facilitate mobility (Remelius et al. 2014; Winter 1995). Our group and others have found that autistic children exhibited reduced postural sway amplitude and variability when stepping forward or initiating walking (Bojanek et al. 2020; Fournier et al. 2010), highlighting altered postural control when transitioning from static to dynamic tasks in younger ages. Since static and dynamic stance are differentially linked to unique neurophysiological mechanisms of postural control, it is imperative to quantify postural sway variability across both types of stance in middle aged and older autistic adults.

Autistic individuals also exhibit atypical coordination during neuromotor activities. For example, autistic children and adolescents show jerkier hip and knee kinematics during non‐constrained walking (Nobile et al. 2011), unstable torso orientation during lateral arm reaching (Miller et al. 2023), and unsteady movement trajectories when swinging their arms horizontally across the torso (Cook et al. 2013). Autistic children also demonstrate jerkier strokes while engaging in handwriting or drawing activities compared to non‐autistic children (Cook et al. 2013; Fuentes et al. 2009). These observations suggest that motor coordination challenges span multiple effector systems and multiple types of behavior in young autistic individuals. Postural control involves simultaneous regulation of anterior–posterior (AP) and mediolateral (ML) sway to stabilize torso movements against self‐ and externally triggered disturbances (Horak 2006; Riccio 1993). Little is known about the modulation of AP and ML postural sway in middle aged and older autistic adults during static and dynamic stance.

Postural development in autistic children tends to occur at a slower rate and remains immature throughout early adulthood (Fears et al. 2023; Lajeunesse et al. 2023; Minshew et al. 2004; Wang et al. 2016; Wolf and Goldberg 1986). Unlike non‐autistic children, who typically exhibit a gradual decline in postural sway variability from early childhood into young adulthood (Cuisinier et al. 2011; Minshew et al. 2004; Verbecque et al. 2016), autistic children maintain relatively consistent postural sway amplitude until early adolescence (Fears et al. 2023; Fournier et al. 2010; Minshew et al. 2004; Wang et al. 2016). Additionally, while non‐autistic individuals generally show greater postural sway variability in the AP direction, autistic children aged 6 and older demonstrate increased sway in the ML direction (Chang et al. 2010; Fournier et al. 2010; Kohen‐Raz et al. 1992; Molloy et al. 2003). This postural sway pattern resembles that of non‐autistic toddlers, who adopt a wider stance and rely on lateral sway to maintain stability at younger ages. A slower reduction in postural sway variability and a persistent reliance on lateral sway for balance highlight underdeveloped postural control mechanisms in autistic youth (Kirshenbaum et al. 2001; Laughton et al. 2003). Despite these well‐established findings, there has been relatively little work to characterize age associated postural sway variability and coordination changes in middle aged and older autistic adults.

The current study aimed to quantify fundamental postural control deviations in middle aged and older autistic adults. We examined the extent to which autistic adults exhibited greater variability and reduced coordination in postural sway patterns during static and dynamic stance relative to non‐autistic adults. We also assessed the effect of age on postural control variables that differentiated autistic adults from non‐autistic adults. Following previous studies in autistic youth, we hypothesized that autistic adults would demonstrate increased COP standard deviation (COP_SD_) in both AP and ML directions and greater COP trajectory length (COP_TL_) relative to non‐autistic adults during static stance, but reduced COP_SD_ in the target direction during dynamic stance. We also expected autistic adults to show jerkier postural sway patterns, greater postural sway range of motion (ROM) variability, and slower sway frequency compared to non‐autistic adults during dynamic stance, demonstrating diminished motor coordination in older age. Next, despite limited research, we predicted that age‐associated postural sway variability would be more prominent in non‐autistic adults while autistic adults would exhibit negligible age‐related postural sway changes. Finally, we explored the relationship between postural control, demographic, and clinical severity measures of ASD.

## 2 Methods

The Institutional Review Board (IRB) at the University of Florida approved all procedures associated with the current study following the Declaration of Helsinki. The IRB number is 202100659, and the approval date was July 26, 2022.

### 2.1 Study Participants

Forty‐seven autistic adults and 48 age‐, sex‐, and full‐scale IQ (fsIQ) matched non‐autistic adults participated in this study (Table 1). Autistic adults were identified through the Center for Autism and Related Disabilities (CARD) at the University of Florida in Gainesville, the University of Central Florida, and the University of South Florida, and SPARK Research Match (https://www.sfari.org/resource/research‐match/). Non‐autistic individuals were primarily recruited from communities in north central Florida through study flyers and word of mouth. After a comprehensive review of the study procedure, all participants provided written informed consent.

**TABLE 1:** Demographic characteristics of autistic and non‐autistic adults.

	ASD	Non‐ASD	t/*χ* ^2^	*p*
	(Mean ± SD)	(Mean ± SD)		
Sample size (*n*)	47	48	−	−
Age (years)	45.89 ± 11.50	48.46 ± 11.21	−1.100	0.274
Sex (M/F)^a^	25/22	22/26	0.514	0.473
Height (cm)	169.94 ± 9.54	168.94 ± 10.85	0.478	0.634
Weight (kg)	94.00 ± 25.55	80.13 ± 17.12	3.102	0.003**
BMI	32.47 ± 8.02	27.93 ± 4.49	3.391	0.001***
WASI‐II				
fsIQ	105.40 ± 13.06	108.25 ± 11.75	−1.117	0.267
vIQ	106.36 ± 13.84	106.15 ± 10.95	0.084	0.933
pIQ	102.87 ± 12.52	108.52 ± 13.81	−2.087	0.040*
ADOS‐2				
Total raw	10.93 ± 3.38	−	−	−
CS^b^	8.43 ± 2.82			
SBRI^c^	2.48 ± 1.43			
RBS‐R total raw	44.52 ± 28.08	2.60 ± 3.45	10.052	< 0.001***

^a^ Chi‐squared statistics.

^b^ Communication + social interaction (CS).

^c^ Stereotyped behaviors and restricted interests (SBRI).

* *p* < 0.05.

** *p* < 0.01.

*** *p* < 0.001.

Prospective autistic adults with a clinical diagnosis of ASD were initially screened using the Autism Spectrum Quotient for Adults (AQ) (Allison et al. 2012) and the Social Responsiveness Scale Adult Self‐Report, Second Edition (SRS‐2) (Constantino 2012). Individuals who scored ≥ 32 on AQ or ≥ 65 on SRS‐2 were invited to receive a diagnostic evaluation using the Autism Diagnostic Observation Schedule, Second Edition (ADOS‐2) (Lord et al. 2012). The diagnosis of ASD for autistic adults was confirmed through a comprehensive review of AQ, SRS‐2, ADOS‐2, and expert clinical opinion (AMO and RAR) following DSM‐5 criteria (American Psychiatric Association 2013). Autistic adults were excluded if they had a known genetic or metabolic disorder associated with ASD (e.g., Fragile X syndrome, Rett syndrome, tuberous sclerosis, etc.). Prospective non‐autistic individuals who scored < 22 on AQ and < 60 on SRS‐2 were enrolled in the study. Non‐autistic individuals were excluded if they reported a family history of ASD in their 1st‐ or 2nd‐degree relatives.

Autistic and non‐autistic individuals who met at least one criterion below were excluded from the current study: (1) had a fsIQ < 75 as measured using the Wechsler Abbreviated Scales of Intelligence, Second Edition (WASI‐II) (Wechsler 2011), (2) confirmed diagnosis of mild cognitive impairment, Alzheimer's disease, or any form of dementia; (3) confirmed diagnosis of non‐specific developmental delay; (4) recent history of or current psychiatric conditions (e.g., schizophrenia); (5) recent history of or current medical illness that significantly affects the structure and/or function of the central nervous system (e.g., brain tumor, thyroid disease, Cushing's disease, or HIV infection); (6) recent history of or current musculoskeletal conditions affecting balance and postural control (e.g., stroke, multiple sclerosis, or osteoarthritis); (7) confirmed diagnosis of a neurological disorder (e.g., seizure disorders, cerebral palsy, Parkinson's disease, or cerebellar ataxia); (8) family history of a hereditary neurological disorder (e.g., Huntington's Chorea or Wilson's Disease); (9) non‐English speaking; or (10) pregnant.

Nine autistic adults reported a history of concussion resulting from risky play during childhood or car collisions in adulthood. For safety reasons, autistic adults were not required to withhold routine psychotropic medications prior to testing, as many traveled independently to Gainesville. Medication use within 48 h prior to the postural control test was as follows: antipsychotics/neuroleptics (ASD = 4), antidepressants (ASD = 31, non‐ASD = 3), antihypertensives (ASD = 24, non‐ASD = 11), sedatives/hypnotics/anxiolytics (ASD = 11), stimulants (ASD = 5), and anticonvulsants (ASD = 8).

### 2.2 Apparatus and Procedures

All postural control tasks were completed with participants standing barefoot on a Qualisys (Qualisys Inc., Buffalo Grove, IL) strain gauge force platform (60.0 × 40.0 cm^2^) at a sampling rate of 960 Hz. Prior to testing, a tracing paper was placed over the force plate. Participants were requested to stand comfortably on the paper with their feet approximately shoulder‐width apart. Participants' feet were traced afterward to ensure they returned to the same position for each trial.

The postural control test consisted of a static stance and two dynamic postural sway conditions. For static stance trials, participants were instructed to rest their arms on the sides and stand as still as possible on the force plate. During dynamic AP sway, participants were requested to primarily use their ankle joints to sway forward and backward continuously on the plate without lifting their toes and heels. For dynamic ML sway, all instructions were the same as the AP condition except for the emphasis on postural sway being modulated through the hip joints sideways. We requested participants to sway in the target direction at a comfortable speed and amplitude for both dynamic tasks. Participants were also instructed to keep their eyes open with their heads oriented forward for the duration of each trial across all conditions. Our research coordinators (DJS and HMG) demonstrated every task after providing participants with the instructions to ensure comprehension. We also requested participants to practice each task before data acquisition to allow for any instruction clarifications and foot position adjustments.

Each condition involved three 45‐s trials administered as a block. Because previous studies have shown that the after‐effect of dynamic trials amplified postural sway variability of static stance (Chiba et al. 2016; Coelho and Teixeira 2017; Stoffregen et al. 2000), our test always began with the static stance followed by dynamic trials. Dynamic conditions were pseudo‐randomized for each individual. Data acquisition was initiated approximately 5‐s after participants initiated the stance (e.g., participants have started swaying on the plate) to avoid collecting the transitioning phase of the COP data following our previous procedures (Bojanek et al. 2020; Wang et al. 2016). To avoid fatigue, breaks were offered between trials and additional breaks were available upon request.

### 2.3 Data Post‐Processing and Analysis

The center of pressure (COP) time series were collected from the force plate to quantify individuals' postural sway. All COP time series were processed and analyzed using custom scripts in MATLAB 2024a (MathWorks Inc. Natick, MA). To minimize variable effects relating to postural stance initiation and fatigue at the end of a trial, the transitioning COP trajectory (represented as a “tail” in the data) at the beginning or the end of a trial was inspected and cropped when present. The remaining COP time series in the AP (COP_AP_) and ML (COP_ML_) directions were down‐sampled to 120 Hz and low‐pass filtered using a 4th‐order double‐pass Butterworth filter at a cutoff frequency of 7 Hz.

For static and dynamic trials, we derived the COP standard deviation in the AP (COP_SD‐AP_) and ML (COP_SD‐ML_) directions and trajectory length (COP_TL_) to quantify postural sway variability. The COP_TL_ is a resultant metric that integrates COP_AP_ and COP_ML_ time series of a standing condition to quantify the COP excursion path on the force plate. The COP_TL_ estimates the sum of the distances between consecutive COP data points over a trial and was calculated following the formula (Bojanek et al. 2020; Wang et al. 2016):

(1) $$\begin{array}{c} \mathrm{COP}\;\text{trajectory length}\;\left({\mathrm{COP}}_{TL}\right)\\ =\sum_{i=1}^{n}\sqrt{{\left({\mathrm{COP}}_{\mathrm{AP}\left(i+1\right)}-{\mathrm{COP}}_{\mathrm{AP}\left(i\right)}\right)}^{2}+{\left({\mathrm{COP}}_{\mathrm{ML}\left(i+1\right)}-{\mathrm{COP}}_{\mathrm{ML}\left(i\right)}\right)}^{2}} \end{array}$$

where *i* represents the time point from 1 to *n* [i.e., *n* = 5400 (45‐s × 120 Hz)].

We also derived dimensionless squared jerk from the COP time series (COP_DSJP_) to quantify the smoothness of individuals' postural sway following a previous study from our group (Wang et al. 2020). *Jerk* represents the rate of change in COP acceleration or the third derivative of the COP displacement with respect to time, where a lower jerk indicates smoother and more coordinated movement (Flash and Hogan 1985; Wolpert et al. 1995). The COP_DSJP_ was calculated using the formula (Wang et al. 2020):

(2) $$\begin{array}{c} \mathrm{COP}\;\text{dimensionless squared jerk normalized}\;\mathrm{by}\;\text{peak velocity}\;\left({\mathrm{COP}}_{DSJP}\right)\\ =\sqrt{\int_{0}^{\mathrm{MT}}\frac{J{\left(t\right)}^{2}}{2}\mathrm{dt}\;\left(\frac{{\mathrm{MT}}^{3}}{V_{\text{peak}}^{2}}\right)} \end{array}$$

where MT represents the trial duration (i.e., 45‐s), $J\left(t\right)$ is the second derivative of a COP time series, and $V_{\text{peak}}$ is the peak velocity of the COP time series.

For dynamic stance, we also derived postural **s**way frequency (COP_Freq_), average COP range of motion (ROM_avg_), and coefficient of variation of the COP range of motion (ROM_cv_) in the target direction (i.e., COP_AP_ for dynamic AP sway, COP_ML_ for ML sway) for both groups. We employed a peak detection algorithm (Billauer 2015) to identify discrete sway cycles of a trial, where the COP range of motion must exceed 30% of the global COP range (Wang et al. 2016). The COP_Freq_ was calculated as the total number of sway cycles within a trial. The ROM_avg_ represents the mean COP range of motion across all discrete cycles, while ROM_cv_ is the ratio of the standard deviation of the COP range of motion (ROM_SD_) to ROM_avg_ within a trial. Since ML sway typically exhibits greater amplitudes than the dynamic AP condition, ROM_cv_, a normalized variability measure, enables a direct comparison between these two conditions. All postural control variables were averaged across three trials per condition prior to between‐group comparisons.

### 2.4 Statistical Analysis

Demographic characteristics were compared between autistic and non‐autistic individuals using an independent *t*‐test for continuous variables and a chi‐squared test for categorical variables.

Prior to inferential statistical analyses, we applied the Shapiro–Wilk test to assess the normality of all outcome variables. 72% of the COP measures violated the assumption of normality. Therefore, we applied a log_10_ transformation to all COP variables. To examine COP_SD_ differences between autistic and non‐autistic individuals, we conducted a 3 stance condition (static vs. dynamic AP sway vs. dynamic ML sway)× 2 COP direction (AP vs. ML) × 2 group (ASD vs. non‐ASD) analysis of covariance (ANCOVA) with weight included as a covariate. In this model, stance condition and COP direction were within‐subject factors and group was a between‐subject factor. We also conducted two separate 3 stance condition × 2 group fixed effects ANCOVAs to assess COP_TL_ and COP_DSJP_ differences between the two groups. Stance condition was included as a within‐subject factor, and group was entered as a between‐subject factor in these models. For dynamic conditions, we compared differences in ROM_avg_, ROM_cv_, and COP_Freq_ using a series of 2 stance condition × 2 group ANCOVAs. Bonferroni adjustment was applied to minimize Type I errors in these ANCOVA models.

A series of multilinear regression analyses were applied to examine the age effect on COP measures found to be different between the two groups. Each regression model consisted of group, age, and group × age interaction as predictors and a COP variable as the response. Lastly, Spearman's rho correlation was applied to examine the relationships between COP variables found to be different between groups and demographic characteristics of ASD, including IQ, RBS‐R, and ADOS‐2 scores. Correlation findings were adjusted for each combination of group and demographic measure using the false discovery rate (FDR) correction (Benjamini and Hochberg 1995).

Statistical analyses were conducted using SPSS (IBM Corp. Released 2021. IBM SPSS Statistics for Windows, Version 28.0. Armonk, NY: IBM Corp). All findings were interpreted as significant at *p* < 0.05.

## 3 Results

### 3.1 Demographic Characteristics

Autistic and non‐autistic individuals were matched on age [*t*(93) = −1.100, *p* = 0.274], sex [*χ* ^2^(1) = 0.514, *p* = 0.473], height [*t*(93) = 0.477, *p* = 0.634], verbal IQ [vIQ; *t*(93) = 0.084, *p* = 0.933], and fsIQ [*t*(93) = −1.117, *p* = 0.267] at the group level (Table 1). Autistic adults had lower performance IQ [pIQ; *t*(93) = −2.087, *p* = 0.040] and greater body weight [*t*(80.181) = 3.102, *p* = 0.003] and body mass index [BMI; *t*(71.924) = 3.391, *p* = 0.001] compared to non‐autistic adults. Autistic adults also exhibited greater RBS‐R total raw scores relative to non‐autistic adults [t(46.302) = 10.052, *p* < 0.001]. See Table S1 for demographic comparisons at the individual level.

### 3.2 Qualitative Characterization of Task Performance

All autistic adults completed postural control tasks without using assistive devices. Twelve autistic individuals were unable to comply fully with task instructions. Specifically, these participants talked or hummed (*n* = 3), swung hands forward and backward (*n* = 1), rested hands on hips or in pockets (*n* = 2), or wore socks (*n* = 1) during the entire testing session. Three autistic adults were unable to keep their gaze forward during most of the trials. Three additional autistic adults slightly raised their toes or heels, and two autistic adults held their arms out to the side for balance during dynamic stance.

**FIGURE 1:**
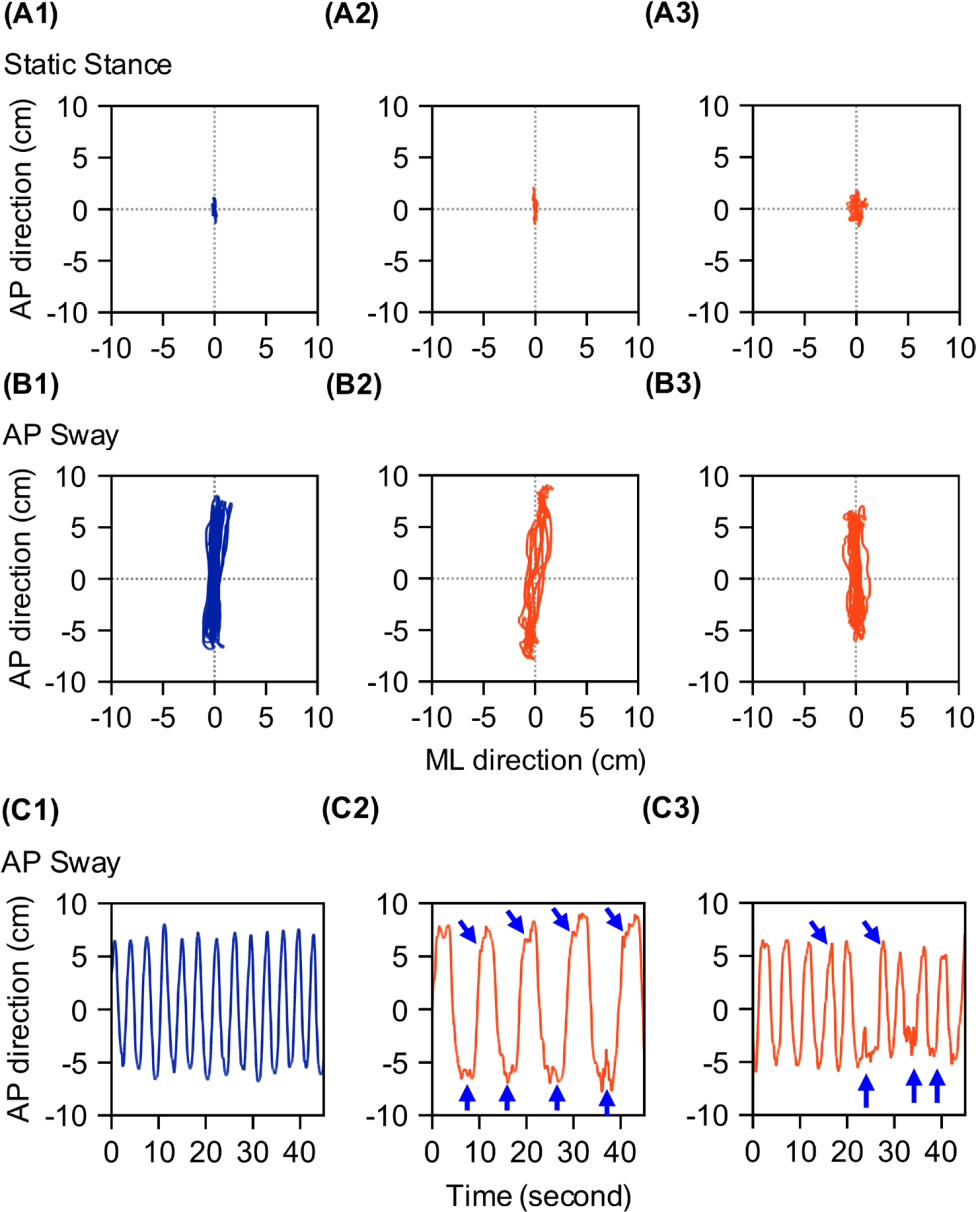
Representative center of pressure (COP) trajectories of a 47‐year‐old non‐autistic male (left), a 42‐year‐old autistic male (middle), and a 43‐year‐old autistic female (right) during static stance (top) and dynamic anterior–posterior postural sway (middle and bottom). The top and middle panels show COP patterns in two dimensions (2D). The bottom panel shows the COP time series in the target direction (i.e., COP_AP_). Blue arrows point at the turning point of sway cycles where autistic adults showed jerkier COP trajectories compared to the control individual.

Figure 1 illustrates representative COP trajectories of one non‐autistic (left column) and two age‐matched autistic adults (middle and right columns) during static stance and AP postural sway. During static stance, the non‐autistic individual exhibited a tightly centered COP pattern (Figure 1A1), while the autistic adults showed more dispersed COP trajectories in both directions (Figure 1A2,A3). The two autistic participants also exhibited jerkier sway relative to the non‐autistic participant during dynamic stance (Figure 1B1–B3), with notably less smooth trajectories around the turning points of sway cycles (blue arrows in Figure 1C1–C3).

### 3.3 Postural Control Deviations in Autistic Adults

**FIGURE 2:**
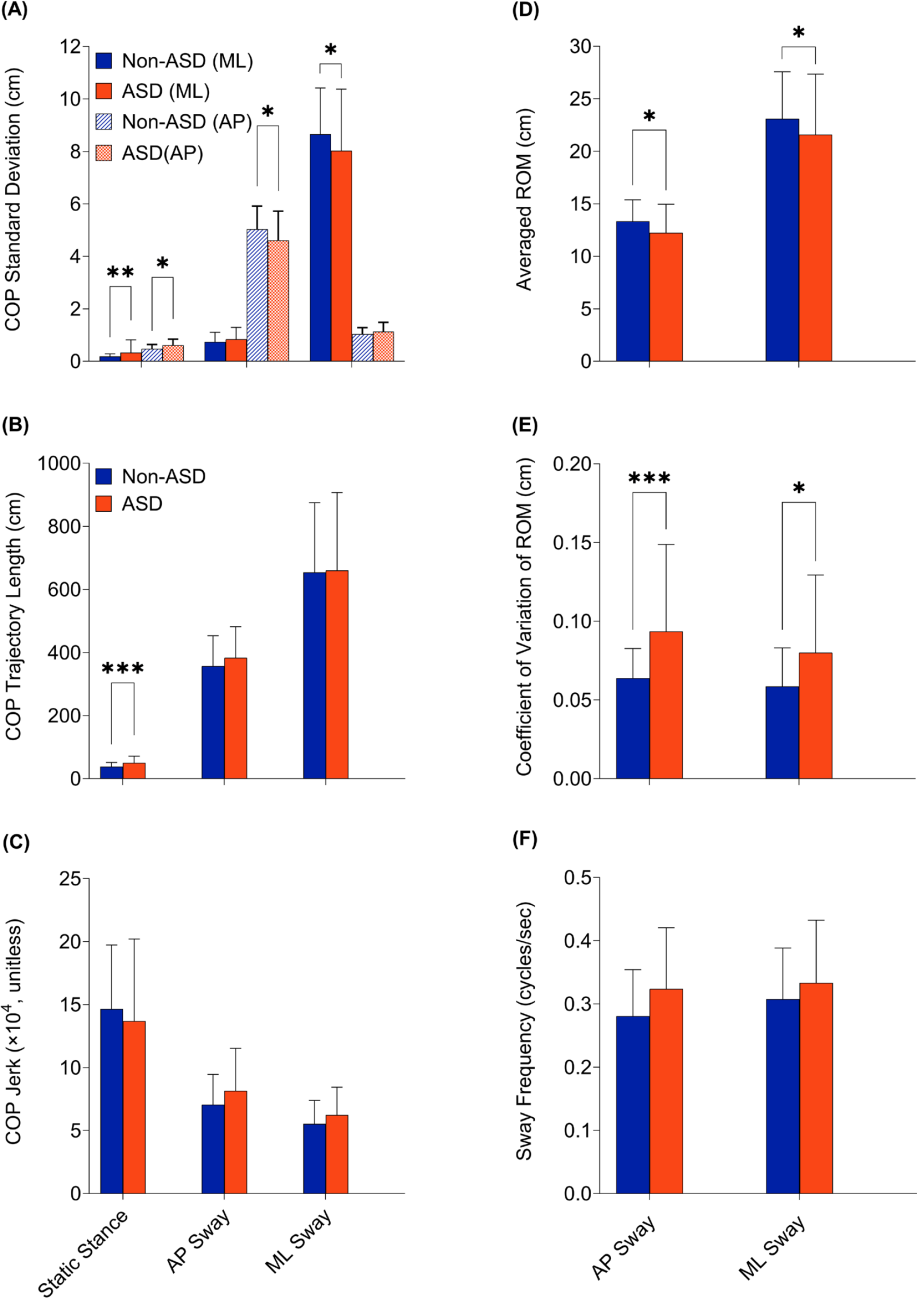
Bar graphs demonstrate the results of repeated‐measures ANOVAs. (A) COP standard deviation (COP_SD_); (B) COP trajectory length (COP_TL_); (C) dimensionless squared jerk normalized by peak velocity (COP_DSJP_); (D) averaged COP range of motion (ROM_avg_); (E) coefficient of variation of COP range of motion (ROM_cv_); (F) postural sway frequency (COP_Freq_). Between‐group differences are denoted as * when *p* < 0.05, ** when *p* < 0.01, and *** when *p* < 0.001. Error bars represent standard deviations.

The COP_SD_ was significantly greater during dynamic stance compared to static stance [stance condition main effect: *F*(1.529, 139.156) = 112.864, *p* < 0.001]. A significant three‐way interaction was observed among group, stance condition, and COP direction [*F*(1.801, 163.888) = 5.550, *p* = 0.006]. Specifically, autistic adults demonstrated greater COP_SD_ than non‐autistic adults in both directions during static stance (COP_SD‐AP_: ASD − non‐ASD = 0.152 cm, SE = 0.056, *p* = 0.008; COP_SD‐ML_: ASD − non‐ASD = 0.073 cm, SE = 0.034, *p* = 0.034; Figure 2A). In contrast, autistic individuals exhibited significantly lower COP_SD_ in the target direction during dynamic stance (COP_SD‐AP_ during AP sway: ASD − non‐ASD = −0.049 cm, SE = 0.023, *p* = 0.032; COP_SD‐ML_ during ML sway: ASD − non‐ASD = −0.053 cm, SE = 0.026, *p* = 0.046). No significant between group differences were found in COP_SD_ for the direction orthogonal to the target during dynamic stance (COP_SD‐ML_ during AP sway: ASD − non‐ASD = 0.069 cm, SE = 0.038, *p* = 0.072; COP_SD‐AP_ during ML sway: ASD − non‐ASD = 0.030 cm, SE = 0.026, *p* = 0.252).

The COP_TL_ was greater for the dynamic stance compared to the static stance [stance condition main effect: *F*(1.389, 126.399) = 146.132, *p* < 0.001]. Autistic adults exhibited greater COP_TL_ relative to non‐autistic adults, though this between‐group difference was non‐significant across conditions [group × stance interaction: *F*(1.389, 126.399) = 2.755, *p* = 0.086; Figure 2B]. Pairwise comparisons revealed that autistic adults demonstrated significantly greater COP_TL_ during static stance (ASD − non‐ASD = 0.093 cm, SE = 0.032, *p* = 0.004), but the two groups did not differ during dynamic stance (AP sway: ASD − non‐ASD = 0.039 cm, SE = 0.026, *p* = 0.131; ML sway: ASD − non‐ASD = −0.017 cm, SE = 0.035, *p* = 0.636).

The COP_DSJP_ was greater during static stance compared to dynamic conditions [stance condition main effect: *F*(1.544, 140.467) = 25.307, *p* < 0.001]. Compared to non‐autistic individuals, autistic adults exhibited lower COP_DSJP_ during static stance, but greater COP_DSJP_ during dynamic stance [group × stance interaction: *F*(1.544, 140.467) = 7.983, *p* = 0.001; Figure 2C]. Pairwise comparisons of group× stance interaction were non‐significant.

Compared to dynamic ML sway, the ROM_avg_ was lower and the ROM_cv_ was greater for the AP sway task [stance condition main effect: ROM_avg_: *F*(1, 91) = 23.784, *p* < 0.001; ROM_cv_: *F*(1, 91) = 4.620, *p* = 0.034; Figure 2D,E]. No significant group × stance interaction was identified for ROM_avg_ or ROM_cv_. Upon inspecting pairwise comparisons, we found that autistic adults exhibited ROM_avg_ reductions (AP sway: ASD − non‐ASD = −0.053 cm, SE = 0.021, *p* = 0.012; ML sway: ASD − non‐ASD = −0.052 cm, SE = 0.024, *p* = 0.032), but ROM_cv_ increases (AP sway: ASD − non‐ASD = 0.148, SE = 0.038, *p* < 0.001; ML sway: ASD − non‐ASD = 0.108 cm, SE = 0.042, *p* = 0.012) during both dynamic tasks.

The COP_Freq_ did not differ between autistic and non‐autistic adults [group main effect: *F*(1, 91) = 0.009, *p* = 0.924]. No significant effects were found for stance condition [*F*(1, 91) = 1.686, *p* = 0.197] or the group × stance interaction [*F*(1, 91) = 0.527, *p* = 0.470; Figure 2F].

### 3.4 Age Effect on Postural Control Deviations in Autistic Adults

Multilinear regression analyses were applied to assess age and group × age interaction on COP variables that differentiated autistic adults from non‐autistic adults (Figure S1). All regression models were significant except for the last model predicting ROM_cv_ during dynamic ML sway (Table 2). Group was a significant predictor for COP_SD‐AP_, COP_SD‐ML_, and COP_TL_ during static stance. The group × age interaction was also significant in predicting COP_SD‐AP_ during statitic stance. Age did not predict any COP variables.

**TABLE 2:** Multilinear regression results of COP variables and predictive values for group, age, and group × age interaction.

COP variables	Model			Group			Age		
	Adj.*R* ^2^	F	*p*	Std. β	*t*	*p*	Std. β	*t*	*p*
Log_10_(COP_SD‐AP_) dur. static stance	0.113	4.977	0.003**	−1.217	−2.900	0.005**	−0.410	−1.886	0.062
Log_10_(COP_SD‐ML_) dur. static stance	0.063	3.122	0.030*	−0.892	−2.068	0.041*	−0.247	−1.105	0.272
Log_10_(COP_TL_) dur. static stance	0.168	7.335	< 0.001***	−1.118	−2.753	0.007**	−0.156	−0.739	0.462
Log_10_(COP_SD‐AP_) dur. AP sway	0.099	4.426	0.006**	0.459	1.084	0.281	−0.181	−0.825	0.411
Log_10_(ROM_avg_) dur. AP sway	0.083	3.852	0.012*	0.503	1.179	0.241	−0.114	−0.516	0.607
Log_10_(ROM_cv_) dur. AP sway	0.122	5.335	0.002**	−0.691	−1.656	0.101	−0.072	−0.333	0.740
Log_10_(ROM_cv_) dur. ML sway	0.039	2.272	0.085	−0.049	−0.113	0.910	0.062	0.274	0.785

COP variables	Group × age		
	Std. β	*t*	*p*
Log_10_(COP_SD‐AP_) dur. static stance	1.071	2.225	0.029*
Log_10_(COP_SD‐ML_) dur. static stance	0.735	1.488	0.140
Log_10_(COP_TL_) dur. static stance	0.876	1.880	0.063
Log_10_(COP_SD‐AP_) dur. AP sway	−0.248	−0.510	0.611
Log_10_(ROM_avg_) dur. AP sway	−0.275	−0.562	0.575
Log_10_(ROM_cv_) dur. AP sway	0.366	0.765	0.446
Log_10_(ROM_cv_) dur. ML sway	−0.240	−0.480	0.633

Abbreviations: Adj.*R* ^2^: adjusted *R* ^2^; Std. β: standardized coefficient beta.

* *p* < 0.05.

** *p* < 0.01.

*** *p* < 0.001.

### 3.5 Demographic Characteristics and COP Variables

IQ scores were not associated with any COP measures in non‐autistic individuals (all *p*s > 0.05; Table S2). In autistic adults, lower vIQ scores were linked to greater ROM_cv_ during dynamic AP sway (rho = −0.413, *p*_FDR_ = 0.032; Table S3). No significant correlations were found between RBS‐R or ADOS‐2 total raw score and any COP measures in autistic adults (all *p*_*FDR*s_ > 0.05; Table S4).

## 4 Discussion

Neuromotor dysfunction is common in young autistic individuals and appears to persist into and throughout adulthood (Bhat 2020; Fournier et al. 2010; Starkstein et al. 2015). However, empirical studies systematically quantifying neuromotor control and coordination impairments in autistic adults, particularly during middle to older adulthood, are limited (Cho et al. 2022; Linke et al. 2018). Our study aimed to examine postural control deviations in autistic adults aged 30–73 using standard static stance and rarely implemented dynamic postural sway paradigms. We report five key findings. *First*, autistic adults exhibited greater COP_SD‐AP_, COP_SD‐ML_, and COP_TL_ than non‐autistic adults during static stance (Figure 2A,B). *Second*, during dynamic stance, autistic adults exhibited decreased COP_SD_, reduced ROM_avg_, but increased ROM_cv_ in the target direction relative to non‐autistic adults (Figure 2A,D,E). *Third*, compared to non‐autistic counterparts, autistic adults demonstrated lower COP_DSJP_ (smoother sway) during static stance but greater COP_DSJP_ (jerkier sway) during dynamic stance (Figure 2C). *Fourth*, during static stance, autistic adults exhibited lower COP_SD‐AP_ in older age, while non‐autistic adults showed greater COP_SD‐AP_ with increasing age (Table 2 and Figure S1A). *Fifth*, greater ROM_cv_ in the target direction during dynamic AP sway was associated with lower vIQ scores in autistic adults (Table S3). Together, these results highlight persistent postural control alterations in autistic individuals across the adult lifespan. Deviations in static and dynamic stance are associated with unique profiles of postural control impairments in autistic individuals. Specifically, increased postural sway variability (i.e., COP_SD_) is more prominently observed during static stance, while compromised motor coordination (i.e., jerkier COP trajectories) characterizes deviations during dynamic stance in autistic adults.

### 4.1 Postural Sway Variability and Coordination Differences in Autistic Adults

Consistent with studies in autistic children and adolescents (Fournier et al. 2010; Minshew et al. 2004; Wang et al. 2016), our data demonstrated greater COP standard deviation (i.e., COP_SD‐AP_ and COP_SD‐ML_) and trajectory length (COP_TL_) in autistic adults during static stance, highlighting increased postural sway variability during tasks that demand stability and minimum range of movement in ASD. Autistic adults also exhibited lower COP_SD_ and ROM_avg_ but a greater range of motion variability (i.e., ROM_cv_) in the target direction relative to non‐autistic adults during both dynamic AP and ML sway. This observation aligns with a study showing increased ROM variability in autistic children when requested to control a moving target displayed on a screen through lateral body sway (Miller et al. 2023). The study of note also found that autistic children preferred to take lateral steps rather than lean their torso to the same extent as non‐autistic children when “moving” the target, suggesting reduced torso movement and reliance on alternative strategies (i.e., taking steps) to achieve the task goal (Miller et al. 2023). As the task goal of dynamic stance was to shift individuals' postural sway toward the postural limitation boundary, reduced COP_SD_ and ROM_avg_ in the target direction reflect a less efficient but more atypical postural control strategy adopted by autistic adults (Fournier et al. 2010; Miller et al. 2023). Notably, decreased postural sway in the target direction has also been observed in non‐autistic elderly during dynamic stance and is strongly associated with increased risk and frequency of falls (Dominguez 2020; Gerards et al. 2017; Horak et al. 1997; Johansson et al. 2019; Melzer et al. 2004; Ringhof and Stein 2018; Rizzato et al. 2021). Our findings suggest that similar neurophysiological mechanisms may underlie increased fall susceptibility in middle aged and older autistic adults, highlighting the urgent need for targeted interventions to enhance postural stability in this population.

As expected, autistic adults showed lower COP_DSJP_ during static stance but higher COP_DSJP_ during dynamic stance compared to non‐autistic adults. During static stance, the COP time series typically exhibit fractal Brownian noise properties, characterized by jerkier trajectories due to the simultaneous modulation of AP and ML sway (Błaszczyk and Klonowski 2001; Chang et al. 2010; Lim et al. 2017; Ramdani et al. 2009). A reduction in COP_DSJP_ indicates a decoupled modulation of postural sway, suggesting that autistic adults may regulate sway predominantly in one direction rather than integrating multidirectional adjustments. In contrast, dynamic stance typically requires the decoupling of AP and ML sway to generate smooth and rhythmical movement in the target direction (Wang et al. 2016). The observed increase in COP_DSJP_ in autistic adults suggests a compromised ability to effectively decouple these two distinct postural sway components during dynamic stance. Notably, autistic adults exhibited the jerkiest COP trajectories at the turning points of the sway cycles (blue arrows in Figure 1C2,C3). Reversing sway direction requires modulating COP velocity to zero while simultaneously maximizing COP acceleration in the opposite direction to initiate a new sway cycle. Our unique observation indicates that autistic adults struggle to meet these biomechanical constraints, potentially reflecting postural coordination difficulties across AP and ML directions.

The group‐by‐age interaction significantly predicted COP_SD‐AP_ during static stance. Consistent with our hypothesis, non‐autistic adults exhibited an age‐associated increase in COP_SD‐AP_, whereas autistic adults demonstrated COP_SD‐AP_ reductions in older age (Figure S1A). While increased COP_SD_ during static stance is associated with the neuromotor decline in typical aging (Roman‐Liu 2018), the paradoxical reductions in COP_SD‐AP_ among autistic adults may reflect atypical neurophysiological adaptations in ASD. Additionally, autistic adults demonstrated significantly greater COP_SD‐ML_ during static stance compared to non‐autistic adults across the whole age range. This finding aligns with previous studies showing that young autistic individuals exhibit greater lateral sway variability than their non‐autistic counterparts (Chang et al. 2010; Fournier et al. 2010; Kohen‐Raz et al. 1992; Molloy et al. 2003). Combining findings alongside increased COP_DSJP_ during dynamic stance (i.e., difficulties in decoupling AP and ML postural sway), it is possible that autistic adults rely more heavily on lateral sway modulation during postural control, contributing to the observed COP_SD‐AP_ reductions in older age. Collectively, our findings underscore the persistence of postural control deviations in autistic individuals across the lifespan.

### 4.2 Postural Control Deviations and Demographic Features in Autistic Adults

We found that increased ROM_cv‐AP_ during dynamic AP sway was associated with lower vIQ scores in autistic adults. Previous research has identified vIQ as a critical factor affecting postural stability in ASD (Minshew et al. 2004; Travers et al. 2018). For example, autistic individuals with lower vIQs (ages 5–52 years) have shown reduced postural equilibrium scores during conditions involving diminished (e.g., eyes closed) or contradictory sensory input (e.g., standing on an unstable force platform embedded within a virtual reality environment) (Minshew et al. 2004). Similarly, autistic children with lower vIQs have exhibited greater postural sway variability even when standing on a stable surface (Travers et al. 2018). These findings underscore a lifelong association between postural control deviations and intellectual ability in autistic individuals, suggesting the potential involvement of shared neurobiological pathways.

### 4.3 Neural Implications of Postural Control in Autistic Adults

Postural control is modulated by a complex supraspinal network involving sensorimotor cortices and subcortical regions, including the cerebellum, basal ganglia, thalamus, and brainstem (Dijkstra et al. 2020). Postural control deviations found in autistic adults may reflect atypicalities within these regions or disruptions in their connectivity. For example, cerebellar‐thalamo‐cortical and basal ganglia‐thalamo‐cortical networks have been consistently implicated in postmortem and brain imaging studies of ASD (Bauman and Kemper 1985; Kemper and Bauman 1993; Schumann and Nordahl 2011; Subramanian et al. 2017; Takakusaki et al. 2004), both underlying neuromotor difficulties in autistic children (di Martino et al. 2011; Mostofsky et al. 2009; Stoodley 2016). Accumulating evidence has also linked volumetric reductions in the brainstem nuclei to sensorimotor impairments in ASD (Dadalko and Travers 2018; Seif et al. 2021; Surgent et al. 2021; Travers et al. 2015). Given that postural control deviations persist into middle and older adulthood in ASD, and that aging‐associated neurodegeneration affects the same supraspinal system in elderly individuals (Fears et al. 2023; Howlin 2023), our findings suggest that middle aged and older autistic adults may face an elevated risk of postural control deteriorations in older age compared to non‐autistic adults.

## 5 Study Limitations

Our study provides evidence that atypical postural control variability and coordination persist throughout middle and older adulthood in ASD. However, our findings should be interpreted in light of several limitations. First, we excluded autistic adults with comorbid intellectual disability, mild cognitive impairment, or dementia, despite evidence linking these conditions to increased fall risk (Axmon et al. 2019; Hsieh et al. 2012) and accelerated neuromotor decline (Geurts et al. 2022; Starkstein et al. 2015; Vivanti et al. 2021). Second, 66% of autistic adults were taking at least one routine psychotropic medication during the lab visit. While the lifelong effects of psychotropic medications on postural control in autistic adults remain largely unknown, their use may have affected the between‐group differences observed in this study. Third, although the dynamic tasks applied in this study resemble some daily activities (Mancini and Horak 2010; Schärli et al. 2012), they offer less ecological validity than paradigms incorporating dual task conditions (e.g., standing while counting backward) or external perturbations (e.g., movable or tilting force platforms). These alternative approaches could provide deeper insight into cognitive load and compensatory mechanisms involved in postural control. Fourth, the cross‐sectional design minimizes our ability to track postural control changes over time and the relationship between postural control deviations and adaptive daily living skills in aging autistic adults remains unclear. Future research should explore this linkage longitudinally to better inform targeted interventions that serve to enhance independence and quality of life in autistic individuals.

## 6 Conclusions

Our study demonstrated atypical postural control in autistic individuals across the adult lifespan. We provide novel evidence that variability in postural sway and coordination manifests differently in autistic adults during static and dynamic tasks compared to non‐autistic participants. In conjunction with previous research indicating worsened physical health in middle aged and older autistic adults (Fears et al. 2023; Hong 2013), our findings suggest that autistic adults may also face an elevated risk of postural control impairments and a heightened likelihood of falls compared to non‐autistic individuals.

## Author Contributions

Z.W. conceptualized and designed the study. D.J.S. and H.M.G. consented to and screened participants. D.J.S., H.M.G., and Z.W. acquired demographic and behavioral data. A.‐M.O. and R.A.R. confirmed the diagnosis for autistic adults. D.J.S., H.M.G., and Z.W. scored the raw data and entered demographic and behavioral data into the database. J.W., H.Q., and Z.W. conducted statistical analyses. H.Q. prepared graphic presentations and tables of the results. J.W., H.Q., and Z.W. interpreted the results. Z.W. and H.Q. drafted the manuscript. Z.W. and B.A.Z. edited the manuscript. All authors approved the final version of the manuscript.

## Ethics Statement

All procedures involved in this study were approved by the Institutional Review Board (IRB) at the University of Florida following the Declaration of Helsinki. The IRB number is 202100659, with an approval date of July 26, 2022.

## Consent

All authors have read and approved the submission.

## Conflicts of Interest

The authors declare no conflicts of interest.

## Supporting information


Data S1.

## Data Availability

All data are available from the corresponding author upon reasonable request.
