# Supplementary material for: Atypical Postural Control Variability and Coordination Persist Into Middle and Older Adulthood in Autism Spectrum Disorder
Source: Autism Res. 2025 Mar 18;18(4):752–64. doi: 10.1002/aur.70024 (PMC12015802; doi:10.1002/aur.70024)
Supplement: Supplementary file 1 — Data S1. [file AUR-18-752-s001.docx]

# **SUPPLEMENTARY MATERIALS**

## **Figure S1.**


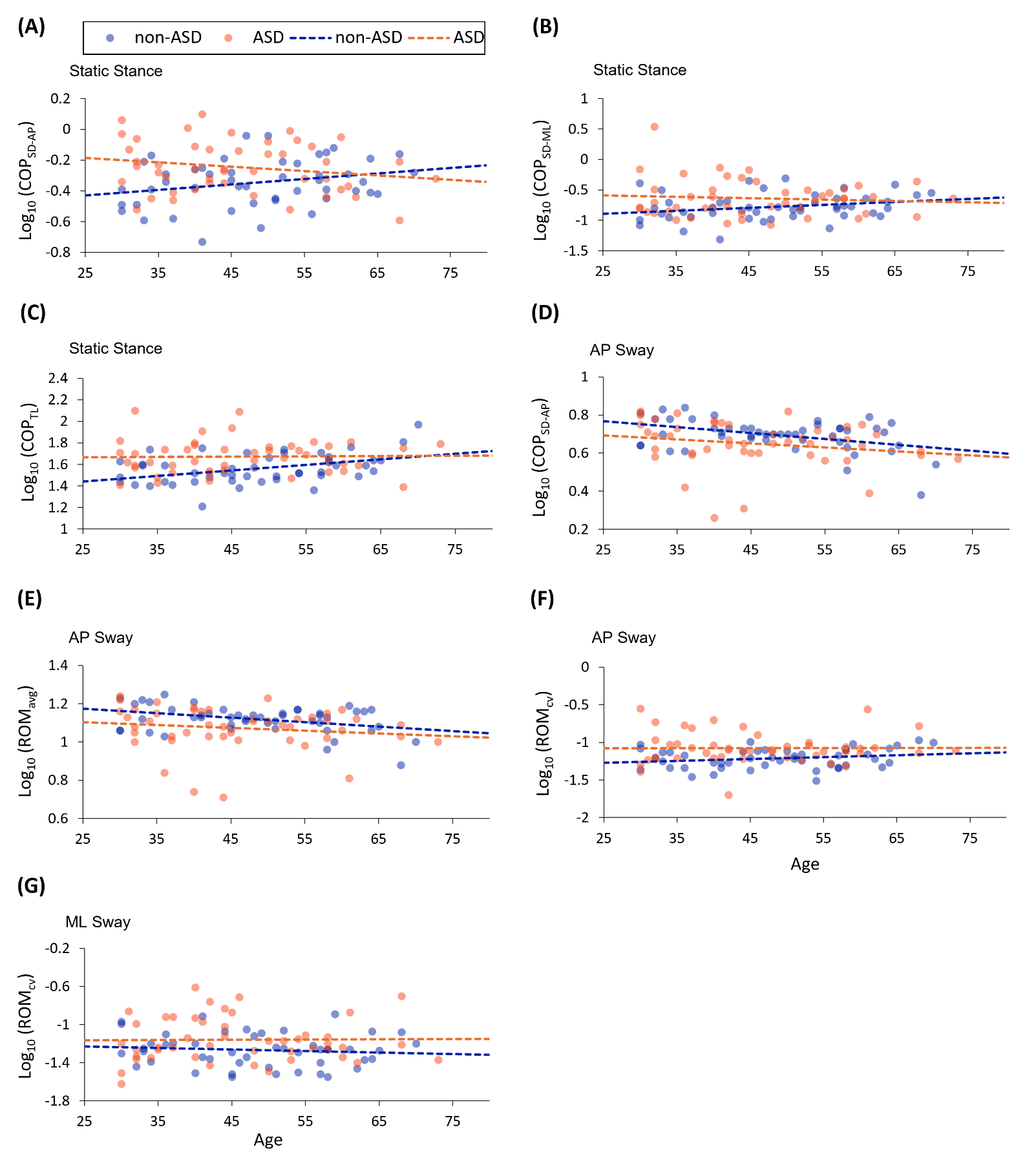


**Figure S1.** Scatter plots demonstrate age-by-group interaction on COP variables (Blue – non-autistic individuals and Orange - autistic adults). (A) Log_10_ (COP_SD-AP_) during static stance; (B) Log_10_ (COP_SD-ML_) during static stance; (C) Log_10_ (COP_TL_) during static stance; (D) Log_10_ (COP_SD-AP_) during dynamic AP sway; (E) Log_10_ (ROM_avg_) during AP sway; (F) Log_10_ (ROM_cv_) during AP sway; (G) Log_10_ (ROM_cv_) dynamic ML sway.

## **Table S1.** Detailed demographic characteristics of autistic and non-autistic adults

|  | **Age** | | **Sex** | | **vIQ** | | **pIQ** | | **Handedness** | |
| --- | --- | --- | --- | --- | --- | --- | --- | --- | --- | --- |
| **Participant** | ASD | non-ASD | ASD | non-ASD | ASD | non-ASD | ASD | non-ASD | ASD | non-ASD |
| 1 | 30 | 30 | M | M | 120 | 95 | 112 | 87 | R | R |
| 2 | 30 | 30 | F | M | 123 | 127 | 105 | 105 | L | R |
| 3 | 30 | 30 | M | M | 71 | 110 | 89 | 107 | R | L |
| 4 | 31 | 32 | F | M | 103 | 115 | 106 | 113 | R | R |
| 5 | 32 | 33 | M | M | 76 | 96 | 95 | 109 | R | R |
| 6 | 32 | 33 | M | M | 102 | 88 | 120 | 134 | R | R |
| 7 | 32 | 34 | F | M | 116 | 99 | 120 | 91 | R | R |
| 8 | 32 | 34 | F | M | 102 | 130 | 88 | 105 | R | R |
| 9 | 34 | 36 | M | M | 122 | 85 | 112 | 109 | R | R |
| 10 | 35 | 36 | M | M | 88 | 93 | 72 | 79 | R | R |
| 11 | 35 | 37 | M | F | 105 | 110 | 101 | 126 | R | R |
| 12 | 36 | 40 | M | F | 103 | 112 | 89 | 100 | R | R |
| 13 | 37 | 40 | F | F | 85 | 115 | 83 | 105 | R | L |
| 14 | 37 | 41 | M | F | 116 | 121 | 96 | 116 | R | R |
| 15 | 39 | 41 | F | F | 131 | 106 | 103 | 111 | R | R |
| 16 | 40 | 42 | F | F | 126 | 108 | 117 | 108 | L | R |
| 17 | 40 | 44 | M | M | 103 | 118 | 103 | 114 | R | R |
| 18 | 40 | 45 | M | F | 123 | 93 | 110 | 87 | R | R |
| 19 | 41 | 45 | F | F | 107 | 95 | 101 | 81 | R | R |
| 20 | 42 | 45 | F | F | 97 | 91 | 95 | 105 | R | R |
| 21 | 42 | 46 | F | M | 101 | 83 | 91 | 86 | R | R |
| 22 | 42 | 47 | M | M | 110 | 103 | 120 | 137 | R | R |
| 23 | 44 | 47 | F | M | 98 | 90 | 120 | 90 | R | R |
| 24 | 44 | 48 | M | M | 131 | 111 | 97 | 116 | R | R |
| 25 | 44 | 49 | F | F | 116 | 111 | 105 | 124 | R | R |
| 26 | 45 | 50 | F | F | 99 | 108 | 91 | 116 | R | R |
| 27 | 46 | 51 | M | F | 90 | 110 | 97 | 124 | R | R |
| 28 | 48 | 51 | M | F | 103 | 115 | 108 | 108 | R | R |
| 29 | 48 | 52 | M | M | 104 | 119 | 112 | 125 | R | R |
| 30 | 50 | 52 | M | M | 124 | 102 | 103 | 103 | R | R |
| 31 | 50 | 54 | F | F | 102 | 105 | 105 | 108 | R | L |
| 32 | 52 | 54 | F | F | 119 | 117 | 98 | 118 | R | R |
| 33 | 53 | 56 | M | F | 101 | 104 | 118 | 123 | R | R |
| 34 | 53 | 57 | M | F | 81 | 113 | 77 | 126 | R | R |
| 35 | 54 | 57 | M | F | 107 | 107 | 96 | 117 | R | R |
| 36 | 55 | 57 | F | M | 105 | 110 | 121 | 88 | R | R |
| 37 | 56 | 58 | M | F | 110 | 119 | 111 | 107 | R | R |
| 38 | 58 | 58 | F | F | 120 | 97 | 134 | 115 | R | R |
| 39 | 58 | 58 | M | M | 113 | 107 | 87 | 120 | R | L |
| 40 | 58 | 59 | F | F | 103 | 111 | 106 | 103 | R | R |
| 41 | 60 | 61 | F | F | 114 | 117 | 105 | 107 | R | R |
| 42 | 60 | 62 | M | M | 106 | 116 | 100 | 120 | L | R |
| 43 | 61 | 63 | M | M | 98 | 100 | 97 | 102 | L | R |
| 44 | 62 | 64 | M | M | 98 | 96 | 101 | 99 | R | R |
| 45 | 68 | 64 | F | F | 87 | 106 | 92 | 126 | R | R |
| 46 | 68 | 65 | F | F | 118 | 89 | 109 | 87 | R | L |
| 47 | 73 | 68 | F | F | 122 | 109 | 117 | 112 | R | R |
| 48 | -- | 70 | -- | F | -- | 113 | -- | 110 | -- | R |

## **Table S2.** Spearman’s rho correlations between IQ scores and COP variables in non-autistic adults

| **COP variables** | **vIQ** | | | **pIQ** | | | | **fsIQ** | | |
| --- | --- | --- | --- | --- | --- | --- | --- | --- | --- | --- |
|  | ρ | *p*_raw_ | *p*_FDR_ | | ρ | *p*_raw_ | *p*_FDR_ | ρ | *p*_raw_ | *p*_FDR_ |
| Log_10_(COP_SD-AP_) dur. static stance | -0.002 | 0.988 | 0.988 | | -0.058 | 0.697 | 0.999 | -0.074 | 0.615 | 0.999 |
| Log_10_(COP_SD-ML_) dur. static stance | -0.089 | 0.546 | 0.874 | | -0.111 | 0.451 | 0.999 | -0.175 | 0.234 | 0.999 |
| Log_10_(COP_TL_) dur. static stance | 0.007 | 0.964 | 0.999 | | -0.024 | 0.874 | 0.874 | -0.076 | 0.607 | 0.999 |
| Log_10_(COP_SD-AP_) dur. AP sway | 0.102 | 0.492 | 0.984 | | -0.170 | 0.248 | 0.999 | -0.062 | 0.677 | 0.903 |
| Log_10_(ROM_avg_) dur. AP sway | 0.163 | 0.268 | 0.999 | | -0.069 | 0.644 | 0.999 | 0.047 | 0.751 | 0.751 |
| Log_10_(ROM_cv_) dur. AP sway | -0.114 | 0.439 | 0.999 | | -0.063 | 0.672 | 0.999 | -0.112 | 0.449 | 0.999 |
| Log_10_(ROM_avg_) dur. ML sway | -0.120 | 0.415 | 0.999 | | -0.049 | 0.742 | 0.848 | -0.057 | 0.699 | 0.799 |
| Log_10_(ROM_cv_) dur. ML sway | 0.081 | 0.583 | 0.777 | | 0.058 | 0.697 | 0.929 | 0.073 | 0.623 | 0.997 |

## **Table S3.** Spearman’s rho correlations between IQ scores and COP variables in autistic adults

| **COP variables** | **vIQ** | | | | | | | **pIQ** | | | | | **fsIQ** | | | | |
| --- | --- | --- | --- | --- | --- | --- | --- | --- | --- | --- | --- | --- | --- | --- | --- | --- | --- |
|  | ρ | | *p*_raw_ | | *p*_FDR_ | | | ρ | | *p*_raw_ | | *p*_FDR_ | ρ | | *p*_raw_ | | *p*_FDR_ |
| Log_10_(COP_SD-AP_) dur. static stance | 0.163 |  | | 0.273 |  | 0.437 | 0.221 | |  | | 0.135 | 0.360 | 0.223 |  | | 0.131 | 0.349 |
| Log_10_(COP_SD-ML_) dur. static stance | 0.025 |  | | 0.866 |  | 0.866 | -0.036 | |  | | 0.810 | 0.926 | -0.010 |  | | 0.948 | 0.948 |
| Log_10_(COP_TL_) dur. static stance | 0.125 |  | | 0.403 |  | 0.461 | 0.164 | |  | | 0.271 | 0.542 | 0.166 |  | | 0.266 | 0.532 |
| Log_10_(COP_SD-AP_) dur. AP sway | 0.224 |  | | 0.131 |  | 0.349 | 0.105 | |  | | 0.483 | 0.644 | 0.148 |  | | 0.320 | 0.427 |
| Log_10_(ROM_avg_) dur. AP sway | 0.232 |  | | 0.116 |  | 0.464 | 0.142 | |  | | 0.340 | 0.544 | 0.159 |  | | 0.286 | 0.458 |
| Log_10_(ROM_cv_) dur. AP sway | -0.413 |  | | 0.004** |  | 0.032* | -0.243 | |  | | 0.100 | 0.400 | -0.368 |  | | 0.011* | 0.088 |
| Log_10_(ROM_avg_) dur. ML sway | 0.156 |  | | 0.296 |  | 0.395 | 0.016 | |  | | 0.916 | 0.916 | 0.091 |  | | 0.542 | 0.619 |
| Log_10_(ROM_cv_) dur. ML sway | -0.167 |  | | 0.261 |  | 0.522 | -0.284 | |  | | 0.053 | 0.424 | -0.274 |  | | 0.062 | 0.248 |

* *p* < 0.05, ** *p* < 0.01

## **Table S4.** Spearman’s rho correlations between ASD traits and COP variables in autistic adults

| **COP variables** | **ADOS-2 total raw score** | | | | | **RBS-R total raw score** | | | | |
| --- | --- | --- | --- | --- | --- | --- | --- | --- | --- | --- |
|  | ρ | | p_raw_ | | p_FDR_ | ρ | | p_raw_ | | p_FDR_ |
| Log_10_(COP_SD-AP_) dur. static stance | 0.137 |  | | 0.363 | 0.726 | 0.091 |  | | 0.546 | 0.999 |
| Log_10_(COP_SD-ML_) dur. static stance | 0.000 |  | | 0.997 | 0.997 | -0.049 |  | | 0.744 | 0.999 |
| Log_10_(COP_TL_) dur. static stance | 0.038 |  | | 0.801 | 0.915 | -0.072 |  | | 0.636 | 0.999 |
| Log_10_(COP_SD-AP_) dur. AP sway | 0.334 |  | | 0.023* | 0.184 | -0.029 |  | | 0.849 | 0.999 |
| Log_10_(ROM_avg_) dur. AP sway | 0.301 |  | | 0.042* | 0.168 | 0.014 |  | | 0.929 | 0.999 |
| Log_10_(ROM_cv_) dur. AP sway | -0.074 |  | | 0.627 | 0.836 | -0.135 |  | | 0.369 | 0.999 |
| Log_10_(ROM_avg_) dur. ML sway | 0.197 |  | | 0.189 | 0.504 | -0.022 |  | | 0.887 | 0.999 |
| Log10(ROMcv) dur. ML sway | -0.132 |  | | 0.382 | 0.611 | -0.073 |  | | 0.628 | 0.929 |

* *p* < 0.05, ** *p* < 0.01

# **GLOSSARY**

**Center of pressure (COP):** The point location of the vertical ground reaction force. The COP time series represents the weighted average of pressure over the surface area (i.e., feet) in contact with the ground. It is commonly used as an indirect measure of postural sway. The COP time series can be derived from the force and moment data collected from a force platform.

**Postural sway variability measures:**

COP standard deviation (COP_SD_): The standard deviation of the COP time series in the anterior-posterior (COP_AP_) or mediolateral (COP_ML_) direction during a standing trial. A higher COP_SD_ indicates greater postural sway variability and reduced stability.

COP trajectory length (COP_TL_): A resultant metric that integrates COP_AP_ and COP_ML_ time series to quantify the total COP excursion path on the force plate during a trial. It represents the sum of the distances between consecutive COP data points. Increased COP_TL_ is associated with greater postural sway variability and instability.

Average COP range of motion (ROM_avg_): The mean value of the COP range of motion in the target direction of a dynamic trial, with a higher ROM_avg_ indicating greater postural sway amplitude.

**Postural coordination measures:**

Coefficient of variation of ROM (ROM_cv_): The ratio of the standard deviation of the COP range of motion (ROM_SD_) to ROM_avg_. A higher ROM_cv_ reflects greater postural sway variability in the target direction during a dynamic trial.

COP dimensionless squared jerk normalized by peak velocity (COP_DSJP_): A metric representing the rate of change in COP acceleration or the third derivative of the COP time series with respect to time. A lower COP_DSJP_ indicates smoother and more coordinated postural sway.

COP frequency (COP_Freq_): The total number of discrete sway cycles during a dynamic trial. A lower COP_Freq_ indicates reduced postural sway frequency and less coordinated postural control.
